# A practitioner's guide to using data on private equity hospital acquisitions

**DOI:** 10.1093/haschl/qxag071

**Published:** 2026-04-07

**Authors:** Sungil Kim, Mark Naslund, Xingzhi Wang, Abid Hasan, Hongbin Huang, Hyong-gu Hwang, Ambar La Forgia, Riley League, Ryan C McDevitt, Kelly Kaili Yang

**Affiliations:** Duke University, Fuqua School of Business, Durham, NC 27708, United States; Duke University, Department of Economics, Durham, NC 27708, United States; Duke University, Department of Economics, Durham, NC 27708, United States; Washington University in St. Louis, Olin School of Business and School of Public Health, St. Louis, MO 63130, United States; Duke University, Department of Economics, Durham, NC 27708, United States; University of Illinois Urbana-Champaign, Gies College of Business, Champaign, IL 61820, United States; University of California, Berkeley, Haas School of Business, Berkeley, CA 94720, United States; University of Illinois Urbana-Champaign, Gies College of Business, Champaign, IL 61820, United States; Washington University in St. Louis, Olin School of Business and School of Public Health, St. Louis, MO 63130, United States; Indiana University, Kelley School of Business, Bloomington, IN 47405, United States

**Keywords:** private equity, hospital acquisitions, data linkage, reproducibility

## Abstract

**Introduction:**

Private equity (PE) investment in US hospitals has attracted substantial policy and research attention, but empirical work has been limited by fragmented and inconsistent transaction data. We aimed to construct a more comprehensive and validated dataset of PE ownership of US hospitals and to provide a practical guide for using these data in research.

**Methods:**

We integrated 6 major commercial deal databases to identify PE investments in US hospitals from 2000 to 2024. We filtered transactions to PE-related hospital deals, matched targets to American Hospital Association (AHA) and the Centers for Medicare & Medicaid Services (CMS) hospital identifiers, manually verified uncertain matches, reconciled duplicate transactions across sources, expanded system-level deals to constituent hospitals, and verified deal and exit dates.

**Results:**

We identified 141 unique PE deals involving 555 unique short-term acute care hospitals, corresponding to 721 hospital-deal observations. The 6 databases differed substantially in deal coverage, deal type, and whether transactions were reported at the hospital or system level. Reliance on a single source would therefore omit many valid deals and could produce biased or incomplete analytic samples. We also found that linking transactions to stable hospital identifiers required substantial manual verification due to system-level transactions, inconsistent reporting, and identifier changes over time.

**Conclusion:**

Accurate study of PE ownership in hospitals requires multisource data construction, transparent validation, and careful linkage to stable hospital identifiers. This harmonized dataset and workflow provide infrastructure for more accurate, transparent, and replicable research on PE ownership in the US hospital sector.

## Introduction

Over the past 3 decades, private equity (PE) firms have acquired ownership stakes in hundreds of US hospitals, sparking a wide policy debate and drawing intense scrutiny from lawmakers regarding the potential for patient harm and hospital closures.^1^ Along with these discussions, a growing body of academic research has examined the consequences of PE investments in hospitals, presenting conflicting evidence of their effects on operational efficiency, financial stability, and patient care.^2-15^

A key limitation of the academic research on PE is the lack of a standardized list of PE transactions, leading researchers to rely on fragmented, costly, and incomplete deal databases. Such databases do not agree on the universe of PE hospital acquisitions, with each source applying different inclusion rules, industry classifications, and reporting conventions.

The lack of a standardized deal list introduces several challenges for researchers. First, when a dataset omits valid deals, researchers may inadvertently classify treated hospitals (ie, those acquired by a PE firm) as controls, leading to measurement error and potential violations of the stable unit treatment value assumption. Second, differences across datasets in the inclusion of certain hospital or deal types can lead researchers to study only a selected—and potentially biased—subsample of PE deals. Third, differences in how hospital information is reported can make it difficult to link hospitals to standard identifiers needed for empirical analysis. Ultimately, the lack of a standardized deal universe means that results may depend on idiosyncratic researcher choices about data sources and sample construction, limiting the reproducibility and comparability of findings across studies.

We address these gaps by constructing a harmonized dataset of PE hospital acquisitions from 2000 to 2024. By integrating 6 major commercial databases, we identify more deals than have been documented previously, finding 141 unique PE deals involving 721 hospital-deal observations across 555 unique short-term acute care hospitals (some hospitals have had multiple transactions). Using our consolidated universe, we show that relying on a single source would only capture a fraction of PE deals and that sources vary in the types of PE deals they include. We also highlight the wide variation in deal lists used in existing research on PE investments in the hospital sector. Finally, we demonstrate the difficulty and importance of matching PE deals to stable hospital identifiers.

To help fill the “missing piece in health care transparency,”^16^ we make our deal list publicly available, release the technical details and code used in our data construction pipeline, and will post updates for any future revisions. Our work will allow researchers to generate their own replicable panels, verify our inclusion criteria, and test the robustness of their results against alternative deal definitions.

## Data and methods

**Figure 1.**
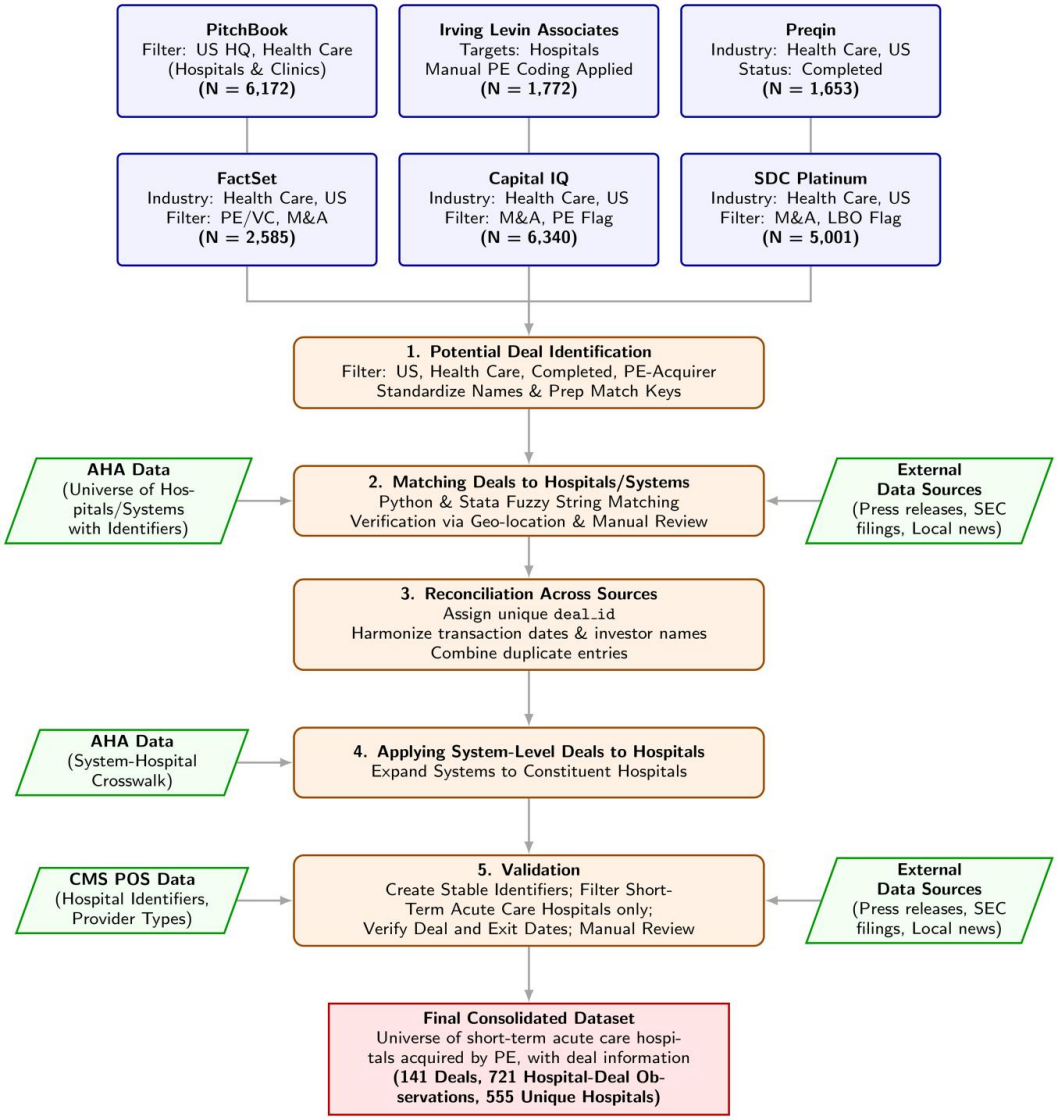
Data Construction Flowchart. This flowchart summarizes the overall data construction workflow used to assemble the hospital-level private equity acquisition dataset. It is intended as a visual guide to the process described in Section 2 and Appendix A.

We construct a more comprehensive deal list of PE investments in short-term acute care hospitals that can be linked to common hospital identifiers. Figure 1 visualizes the data construction process, described below, while Appendix A provides more details.

### Collecting PE deals

To construct the deal list, we draw on 6 major deal databases commonly used in the literature that report PE investments in US hospitals: Irving Levin Associates (Levin), PitchBook, Preqin, Capital IQ, FactSet, and SDC Platinum. Each of these sources applies distinct industry classifications, reporting conventions, and coverage rules. Because all of the sources also contain both non-PE and nonhospital transactions, for each source, we filter to US hospital transactions using variables such as target industry and geography. To limit to PE deals, rather than other merger and acquisition activity, we also filter based on acquirer type. When the acquirer classification is ambiguous, we consult investor descriptions and cross-reference with other databases. In addition, we limit PE deals to leveraged buyouts (LBOs) (including primary, secondary buyouts, and add-on deals), private investment in public equity (PIPE), and growth equity. This restriction excludes real-estate investment trusts (REITs) and joint ventures. Lastly, we collect information on PE exits to classify which years a hospital had a PE investment.

### Matching deals to hospitals

To construct the most comprehensive list possible of PE deals in the hospital sector, we need to map each deal to identifiers that indicate the hospitals and hospital systems involved in each transaction. To build this crosswalk, we rely on data from the American Hospital Association (AHA) annual surveys, which provide the universe of candidate hospitals and systems for linking to deal data.

To conduct this merge, we first standardize hospital and system names as reported in the deal data by removing punctuation, suffixes, and common stopwords and by normalizing abbreviations. We then use fuzzy string matching in both Python (rapidfuzz) and Stata (reclink2) to merge the names of PE deal targets with the list of hospitals and systems from the AHA. This fuzzy match relies on the similarity of the deal data with the reported names in the AHA data, as well as the address, ZIP code, and state. Fuzzy matching generates candidate matches only. We manually review all the candidate matches and resolve uncertain cases using deal descriptions, press releases, U.S. Securities and Exchange Commission (SEC) filings, and local news reports.

### Reconciling sources

After we link each deal listed in each source to their hospital identifiers, we merge the resulting files and reconcile any duplicate representations of the same deal across multiple databases by assigning a unique deal ID and harmonizing reported transaction dates.

### Applying system-level deals to hospitals

Next, we decompose hospital system deals into the hospital level by creating a crosswalk between PE-acquired hospital systems and their constituent hospitals. The AHA annual surveys report system ownership information. When AHA records are missing system affiliation data or reported system hospital counts differ greatly from external sources, we manually map acquired systems to hospitals using deal descriptions, press releases, SEC filings, and local news reports.

### Verifying hospital identifiers

After expanding system-level acquisitions, we assign each hospital a stable identifier to track facilities throughout the panel. We focus on AHA IDs and CMS Certification Numbers (CCNs) because these identifiers are commonly used to link to hospital outcomes. However, standard AHA–CCN crosswalks are frequently incomplete or inaccurate due to administrative missingness, reporting lags, or identifier changes following an acquisition, which can lead to missing links between AHA and CMS hospital identifiers. To address these limitations, we manually verified cases where identifier crosswalks were missing, inconsistent across years, or appeared to change following an acquisition. This verification was based on hospital name, location, and, when necessary, online searches and merger deal documentation. Finally, we restrict the sample to short-term acute care hospitals using CMS provider-type codes from the Provider of Services (POS) file. Specifically, we include hospitals classified by CMS as short-term acute care hospitals and exclude critical access hospitals (CAHs), long-term acute care hospitals, rehabilitation hospitals, and psychiatric hospitals.

### Outputs and reproducibility

The workflow produces an analysis-ready dataset in which each row corresponds to a hospital–deal pair, with stable hospital identifiers across deals, harmonized deal identifiers, source indicators, and verified deal completion and exit dates. We have made the code used in our matching process publicly available and have documented the fuzzy match thresholds, rule-based checks, and manual decisions we used (See https://github.com/sungilkim94/Kim-PE-Data.). Appendix A contains more technical details of our process.

## Findings

We use our dataset to first investigate the discrepancies in PE coverage between the 6 commercial data sources and quantify how relying on a subset of these sources leads to an attenuated sample. Second, we document challenges in linking hospitals identified in PE deals to common hospital identifiers used in the literature, which can also lead to incomplete samples. Third, we compare our harmonized deal list to the number of hospitals included in papers we identified through a literature review of work on PE hospital acquisitions by matching our list as closely as possible to the reported sources and samples used in those papers.

### Comparison across databases

We compare coverage across the 6 PE deal sources and document how overlap patterns and deal definitions differ. Table 1 reports simple counts from each source. The union of sources (our dataset) includes 141 unique PE deals involving 721 hospital–deal observations across 555 unique short-term acute care hospitals, with 124 hospitals involved in multiple transactions. We focus our comparisons on hospital-deal observations (panel B), since this is the most granular level of data available as well as the level needed to conduct empirical analyses and to understand the types of deals associated with each hospital (ie, a hospital may first receive growth equity before undergoing an LBO). As shown in the table, individual sources cover different slices of this universe (eg, PitchBook reports 695 hospitals and 118 deals; Levin reports 416 and 94). Due to overlap, the sum of individual source totals exceeds the total in our final combined dataset.

**Table 1.** Comparison of commercial databases.

	PitchBook	Levin	Preqin	FactSet	SDC	Capital IQ	All sources
**Panel A: An observation is a unique hospital**							
Unique hospital count	544	363	333	348	387	362	555
**Panel B: An observation is a hospital-deal pair**							
Hospital–deal Count	696	416	353	380	439	403	721
*Deal level*							
System level	86.8%	79.1%	90.4%	83.4%	83.4%	80.6%	84.3%
Hospital level	13.2%	20.9%	9.6%	16.6%	16.6%	19.4%	15.7%
*Deal type*							
LBO	70.3%	98.3%	97.7%	98.9%	90.7%	98.2%	71.2%
PIPE	20.8%	0.0%	0.0%	0.3%	6.6%	0.3%	20.1%
Growth equity	8.9%	1.7%	2.3%	0.8%	2.7%	1.5%	8.7%
*Hospital characteristics*							
For-profit	73.3%	70.5%	76.7%	72.0%	73.2%	72.5%	72.3%
Rural	33.8%	32.0%	33.1%	34.3%	36.2%	29.3%	33.4%
Bed count	198	216	216	223	211	217	198
**Panel C: An observation is a deal**							
Deal count	118	94	49	78	84	93	141
*Deal level*							
System level	38.1%	26.6%	44.9%	35.1%	34.5%	31.5%	34.0%
Hospital level	61.9%	73.4%	55.1%	64.9%	65.5%	68.5%	66.0%
*Deal type*							
LBO	83.1%	96.8%	89.8%	94.8%	94.0%	93.5%	85.2%
PIPE	4.2%	0.0%	0.0%	1.3%	1.2%	1.1%	3.5%
Growth equity	12.7%	3.2%	10.2%	3.9%	4.8%	5.4%	11.3%

Panel A reports unique hospital counts across the commercial databases. Panel B reports characteristics at the “hospital–deal” level. This includes hospitals that have been targets in a PE deal more than once. Panel C reports characteristics at the deal, rather than hospital, level. “System level” deals include only deals involving the full hospital system. “Hospital Level” deals include single-hospital and multi-hospital acquisitions by a single buyer (eg, 5 Miami HCA Hospitals). For-Profit: the share of hospitals with for-profit status in the year of acquisition as recorded in the CMS POS file. Rural: the share of hospitals with rural status in the year of acquisition as recorded in the CMS POS file (Note that urban/rural attributes are not available prior to 2011, so only hospitals with deal years 2011-2024 are included in these shares). Bed count: average total bed count among hospitals as recorded in the CMS POS file in the year of acquisition.

**Figure 2.**
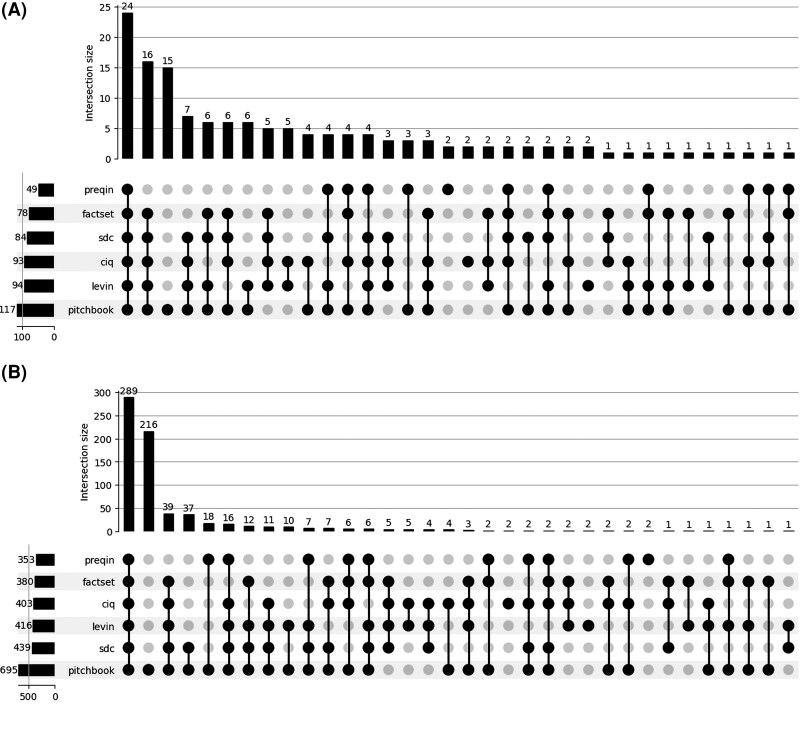
Overlap of Data Sources. This figure presents UpSet plots summarizing overlaps among data sources used to construct the PE hospital acquisition sample from 2000 to 2024. Panel A (Deals) reports intersections at the deal level, where each observation corresponds to a unique transaction. Panel B (Hospitals) reports intersections at the hospital level, where each observation corresponds to an individual hospital involved in a transaction. Bars report the number of deals or hospitals associated with each unique combination of sources, while connected dots indicate which sources contribute to each intersection. Counts are aggregated over the full sample period.

Across the full sample period from 2000 to 2024, coverage overlaps are substantial but incomplete. To visualize the intersections among datasets, Figure 2A and B present UpSet plots of the aggregated data. These figures reveal that, although the full 6-way overlap covers 289 hospitals (24 deals) and the 5-way combination excluding Preqin covers 39 hospitals (16 deals), significant portions of the sample remain source-specific. Notably, PitchBook uniquely identifies 216 hospitals (15 deals) that appear in no other source.

Sources also differ in reporting by “deal level” (ie, whether the deal occurs at the hospital level or system level) and “deal type” (ie, whether the deal involves minority or majority PE ownership), as shown in Table 1. Overall, 66% of deals are at the hospital level, but data from Preqin suggest only 55% and Levin 73%. Highlighting the difference in coverage across data sources, 93% of the hospitals that appear in PitchBook but not Levin are a part of system-level deals, while 85% of those in Levin but not PitchBook are at the hospital level, as shown in Table B2 in Appendix B.

Deal types are assigned to one of 3 categories: LBOs, PIPE, or growth equity. Although LBOs have historically been viewed as the canonical type of PE transaction, commercial databases also capture PIPE and growth equity deals. Each deal type may imply distinct economic mechanisms: LBOs typically involve a change in control and the use of leverage to impose financial discipline,^17^ whereas growth equity and PIPE deals often represent minority stakes focused on capital injection without a full transfer of operational control. Overall, LBO deals account for the largest share (71% of hospital–deal pairs; 85% of deals), while PIPE deals represent 4% of deals covering 20% of hospital acquisitions, and growth equity represents 11% of deals covering 9% of hospital acquisitions. Furthermore, 70% of PE-acquired hospitals had at least 1 LBO, 10% had at least 1 PIPE deal, and 25% had at least 1 growth equity deal, as shown in Appendix B Table BS1. Differences across sources are systematic: 12% of deals in PitchBook but not in Levin are PIPE, and 28% are growth equity, while 100% of those in Levin but not in PitchBook are LBO, as shown in Appendix B Table BS2. Appendix C contains further details regarding deal-type classification. Lastly, sources also differ in the characteristics of the acquired hospitals they cover. Table 1 shows that the share of acquired hospitals that are for-profit ranges from 69% to 76% across sources, with the share of covered hospitals in rural areas ranging from 29% to 36% and average hospital size ranging from 198 to 223 beds. Coverage over time also differs across sources.

Figure BS1 in Appendix B shows that source coverage is more even in earlier years, but PitchBook is the dominant source through the 2010s.

The comparison highlights that although the sources overlap substantially, analyses that rely on a single source may have a biased sample with respect to deal level, deal type, hospital characteristics, and the years in which PE transactions occur. In other words, relying on a limited set of sources can lead to false negatives (eg, treating PE-owned hospitals as controls) and selection bias (eg, capturing only large or publicized deals).

### Challenges in linking PE deals to hospital identifiers

In addition to substantial variation across data sources, linking deals to stable hospital identifiers needed for longitudinal analyses also presents challenges. Three primary difficulties stand out: (1) linking hospitals listed in PE deals to commonly used hospital datasets, (2) expanding system- level deals to constituent hospitals, and (3) tracking hospitals across multiple acquisitions and across datasets.

As discussed in the second section, we developed a rigorous fuzzy matching protocol to first link hospitals from each PE deal source to the AHA data. Despite this process, a total of 48 hospitals (7% of the sample) required manual matching across all included data sources to correct false positive and false negative matches.

Many acquisitions occur at the system level, necessitating expansion of these deals to the hospital level. We use AHA data to expand systems based on AHA system information from the year before the acquisition. Due to known system reporting issues in AHA data^18^; however, we manually assign hospitals to their corresponding system when AHA information is missing or does not align with stated hospital counts in deal descriptions or press releases. A total of 172 hospitals (24% of the sample) are part of system deals that required manual assignment.

Hospital identifiers such as CMS CCNs and AHA IDs can be unstable due to post-acquisition ID changes, administrative missingness, and inconsistencies in how hospitals are reported across AHA and CMS files. To create stable hospital identifiers needed to track hospitals across acquisition events and to resolve inconsistent cases, we manually adjusted identifiers for 24 hospitals (3.3% of the sample).

### Comparison with previous studies

Previous research has generally relied on a subset of these 6 sources for their analysis, as shown in Table 2. For example, Gao, Kim, and Sevilir^9^ start from the hospital merger list created by Cooper et al.^19^ and classify these mergers as PE, while Bhatla et al. use Levin and PitchBook^2^ and Diaz et al.^8^ use data collected by the Private Equity Stakeholder Project Private Equity Hospital Tracker. Table 2 summarizes the different sources that previous studies have used and highlights the variation across them.

**Table 2.** Variation in deal sources and counts across the literature.

	Hospital count^a^			Sources used								
Study	Original	Expanded	Maximum	PB	Levin	Preqin	CIQ	FS	SDC	Zephyr	CBI	PESP^b^
Bhatla et al. (2025)^2^	73	101	200	✓	✓							✓
Bruch, Gondi, and Song (2020)^3^	204	247	392	✓	✓		✓					
Bruch, Zeltzer, and Song (2021)^4^	130	133	133^c^		✓							
Cerullo et al. (2021)^5^	228	240	275	✓						✓	✓	
Cerullo et al. (2022)^6^	184	233	256	✓						✓	✓	
Cerullo et al. (2022)^7^	257	277	339	✓						✓	✓	
Diaz et al. (2025)^8^	67	125	339									✓
Gao, Kim, and Sevilir (2025)^9^	419^d^	352	649	✓		✓	✓	✓	✓			
Kannan, Bruch, and Song (2023)^10^	51	114	200	✓	✓							
Kannan et al. (2025)^11^	49	112	200	✓	✓		✓					
Liu (2022)^13^	838^d^	366	576	✓	✓	✓	✓		✓			
Offodile et al. (2021)^14^	282	282	443	✓						✓	✓	
**This paper**			**721**	✓	✓	✓	✓	✓	✓			

This table compares the counts of hospitals with PE investment across previous studies in the literature.

Abbreviations: CBI, CB Insights; CIQ, Capital IQ; FS, FactSet; PB, PitchBook; SDC, SDC Platinum.

^a^The “Original” column reflects the reported hospital count in each study. Because these counts are in part determined by analytic sample restrictions, we report the unique hospital counts calculated by applying these restrictions (described in Table D2) on our dataset in the “Expanded” column. This “Expanded” column should therefore approximate each study's sample using our dataset. We also report the total possible PE hospital count over the relevant period considered by each study in the “Maximum” column. The values reported in the “Maximum” column are hospital-deal observations rather than unique counts.

^b^The sources in the right-most panel (Zephyr, CBI, PESP) were reviewed but excluded from our primary data construction because they did not provide unique deals beyond our existing primary sources. PESP (Private Equity Stakeholder Project) is a tracker of current hospital ownership that compiles lists from public sources rather than a primary transaction database. SEC filings were sometimes used for deal verification, but are excluded here as they do not constitute a formal transaction database. Our deal list completely subsumes deals reported in Zephyr and CBI in the relevant time periods.

^c^Bruch, Zeltzer, and Song^4^ limit their sample to active PE investments. We use our verified exit dates to determine if PE investments were active in 2018. Exit dates are only tracked for LBOs. PIPE and growth equity deals are not included in these counts.

^d^Gao, Kim, and Sevilir^9^ restrict to AHA-defined general medical and surgical hospitals, while Liu^13^ includes all CMS hospital types (including CAHs, long-term care hospitals, rehabilitation hospitals, and psychiatric hospitals). Our sample instead focuses on CMS-defined short-term acute care hospitals.

Table 2 also demonstrates the extent to which reliance on these different sources affects the number of hospitals included in the analysis. In the “Original” column, we report the number of PE-acquired hospitals reported in each study, while in the “Expanded” column, we report the number of unique PE-acquired hospitals that would be included in the study had it used our expanded dataset, based on the criteria reported in that study. We find that for most studies that limit their sample to short-term acute care hospitals, our more comprehensive dataset would result in a larger sample of PE-acquired hospitals, although some differences are small. Note that even in cases where the counts are similar, the hospitals included in the samples may be different.

Furthermore, because existing studies have varying objectives, they also differ in how they define a “PE deal” (ie, some studies explicitly focus on LBOs, while others do not clearly define their inclusion criteria), sample windows, verification of deal statuses, linkages of ownership changes to hospital identifiers, and analytic sample restrictions. We provide more details on these differences in Appendix D. Table 2 shows the degree to which these choices can limit the sample of hospitals analyzed, where the “Maximum” column reports all PE transactions at the hospital-deal level that occurred during the study's sample window without applying any further restrictions. We emphasize that differences in PE counts across studies can be the result of reasonable research design choices, such as limiting to hospitals with outcome data available or to LBOs. Nonetheless, this table demonstrates the degree to which these decisions reduce the number of transactions included in the study.

## Discussion

Based on our data collection process, we document more PE hospital transactions than have been previously reported. We offer 3 methodological insights for future research. First, to obtain a comprehensive sample of PE acquisitions, researchers must use multiple sources, as no single commercial database captures the full universe of PE activity. If a single source must be used, studies should explicitly acknowledge its specific coverage bias. For example, in comparing 2 frequently used datasets, Levin reliably identifies traditional LBOs and single-facility acquisitions, whereas PitchBook offers broader coverage of system-level transactions and minority growth equity deals. Although neither source is comprehensive on its own, their combined use comes much closer to a complete list of PE transactions. As such, researchers should explicitly test the sensitivity of their results to the sources they use to bind the potential bias introduced by missing deals.

Second, researchers should disclose which deal level and types are included in their analysis, as PE is not a monolithic treatment. For example, PE investment into a large hospital system may have distinct effects compared to investment into an individual hospital. Similarly, LBOs, which imply full operational control and substantial debt, may have different effects than growth equity or PIPE deals, which imply capital injection without full control.

Third, researchers should be careful and transparent in their protocols for matching deals to hospitals. These decisions, in addition to differences in data sources, can lead to differences in which PE deals and hospitals are included in analyses and can hinder replication efforts. In particular, researchers conducting longitudinal analyses should be vigilant about using stable identifiers that survive ownership changes and can track multiple acquisitions of the same hospital over time.

### Limitations

While this harmonized dataset offers a more comprehensive list of PE hospital acquisitions found in 6 major commercial deal databases, we may still miss transactions involving smaller hospitals that do not trigger formal reporting or press releases or are missing from all 6 databases for unknown reasons. Our dataset is also limited to PE acquisitions during our sample period and therefore does not account for management or ownership changes that occurred before 2000 or that do not involve PE, such as non-PE mergers and acquisitions, initial public offerings, for-profit conversions, joint ventures involving PE funds, or REITs, all of which could influence hospital operations. In addition, our study focuses on short-term acute care hospitals, the types of hospitals most commonly studied in the literature, but PE has also invested in specialty facilities, such as psychiatric, rehabilitation, and long-term acute care hospitals, all of which remain an area for future research. Finally, the high degree of accuracy in this dataset is a result of extensive manual verification. While our process ensures reliable, high-quality data, the dataset is not a live, automated feed and will require further manual updating to incorporate future transactions. The release of the standardized workflow and matching keys is intended to facilitate these updates as new data become available.

## Conclusion

This paper addresses a fundamental barrier to the economic analysis of the hospital market: the lack of a reliable, verifiable universe of PE acquisitions. We resolve this issue by drawing on 6 commercial databases and publicly releasing a verified list of PE acquisitions of short-term acute care hospitals. In so doing, we provide the infrastructure necessary for the next generation of studies to rigorously evaluate the consequences of PE ownership in the US hospital sector. Our data offer new granularity by identifying multiple acquisitions of the same hospital over time, recording PE exit events, and distinguishing between hospital- and system-level transactions. Furthermore, our classification of all deal types helps researchers distinguish between the economic mechanisms of majority and minority ownership. By providing a standardized framework for deal identification and data linkage, this work enables future scholarship to investigate how PE investment might influence the long-term clinical and operational trajectories of US hospitals.

## Supplementary Material

qxag071_Supplementary_Data
