# Supplementary material for: A practitioner's guide to using data on private equity hospital acquisitions
Source: Health Aff Sch. 2026 Apr 7;4(4):qxag071. doi: 10.1093/haschl/qxag071 (PMC13089501; doi:10.1093/haschl/qxag071)
Supplement: qxag071_Supplementary_Data [file qxag071_supplementary_data.zip › Appendices.docx]

# Appendix A Data Construction Details

This appendix provides more details on our data construction process outlined in Section [2](#_bookmark0), documenting our data construction process and providing specific details for replicability and to facilitate future work on this topic.

## Collecting Potential Deals

This initial stage pulls raw transactions from each platform and applies basic filters: health care targets (only subsetted to more granular sectors when industry coding is reliable), U.S. deals, completed status, and PE-relevant indicators (if available) to form a broad starting subset for further identification. Except where noted, datasets are pulled through their latest available updates in 2025.

The two broad filters most relevant are deal type (M&A) and industry classification (health care). PitchBook provides a clear classification for PE deals, but other datasets either include PE deals under M&A with only partial PE-relevant flags or provide no dedicated PE indicator at all. While restricting the industry to “hospital-relevant” categories yields a smaller and more targeted subset, we find that hospital facilities are sometimes assigned to other sub-industry codes that do not explicitly reference hospitals.[^2^](#_bookmark25) For this reason, we apply health care as the broad industry filter and defer finer hospital-level identification to the later stage.

**PitchBook** We filter at both company and deal levels. At the company level, we retain U.S. headquarters, healthcare sector, and primary industry codes for clinics or hospitals. At the deal level, we retain completed deals classified as private equity. This yields N = 6,172 deals in total (hospitals, N = 643; clinics, N = 5,529).

**Levin** We filter targets to hospitals (*N* = 1*,* 772). We manually coded deal completion and PE involvement using the acquirer field and the deal description. Deals are classified as PE when the acquirer description explicitly indicates private equity; otherwise, we checked acquirer names against PitchBook universe tags and coded as PE when tagged accordingly.

**Preqin** We keep only completed buyout, public to private, add-on, and growth capital deals in the U.S. and restrict the industry to healthcare and sub-industry to hospitals (N = 1,653).

^2^We also conducted a sanity check outside our “healthcare” filter by scanning all PE deals classified in non- hospital/non-health care industries across datasets using inclusive keywords (e.g., “hospital,” “medical center”). Only a small number of hospital facilities appeared under unexpected industry codes. For example, some were labeled as “buildings and property” in PitchBook. We manually verified these cases and found them to be rare.

**Capital IQ** We keep M&A transactions, use the platform’s PE involvement flag, restrict to

U.S. deals, and limit the target sector to “Healthcare Facilities” and “Managed Healthcare” (*N* = 6*,* 340).

**FactSet** We keep M&A, PE/VC, and public equity offering/private placement transactions, restrict the target RBICS industry-sector to “Healthcare” and “Patient Care”, and further limit to completed deals with U.S. targets (*N* = 2*,* 585).

**SDC Platinum** We use the mergers and acquisitions content set, restrict target macro indus- try to health care and target mid-industry to “Other Healthcare”, “Hospitals”, and “Healthcare Providers & Services (HMOs)”, and restrict the target nation to the U.S. and later use leveraged buyout flags to help identify PE buyout deals (N = 5,001).

## Matching Deals to Hospitals

To link PE targets to AHA entities, we create match keys and then run fuzzy matching. Com- pany names in investment databases often differ from official hospital names, so string cleaning and multi-layer matching are required. We describe the Levin–AHA and PitchBook–AHA link- ages in detail, as they illustrate the typical structure of our matching procedure. The same logic applies to Preqin, Capital IQ, FactSet, and SDC.

**Preparation of match keys.** For AHA, we create a single entity name list consisting of all hospitals and all systems with flags to indicate if the entity is a hospital or system. We retain identifiers and location details such as address, ZIP code, city, and state.

For Levin, the name-extraction step converts ambiguous targets (e.g., “2 Connecticut hos- pitals”, “4 HCA hospitals”) into facility-level match keys. We do this using information from deal descriptions and targeted manual searches of SEC filings, local news, and press releases. We disaggregate multi-facility deals, recover hospital or system names from the description or public sources when needed, and retain location variables. Each resulting row corresponds to one hospital or system.

For PitchBook, we construct match keys separately for the hospitals/inpatient services and clinics/outpatient Ssrvices data subsets. We then trim the hospital sample and filter the clinic sample using named entity recognition and keywording across all relevant fields (target name, target description, etc.), keeping inclusive terms such as “hospital” and “medical center” and excluding terms such as “imaging center” and “dental” to isolate hospital-related entities. We calibrate the keywords and named entity recognition to balance potential false positives and false negatives.

We apply the same name-extraction, keywording, and trimming procedure to Preqin, Capital IQ, FactSet, and SDC. The resulting datasets provide the match-key inputs for subsequent fuzzy matching to AHA.

**Fuzzy match programming** We implement fuzzy matching in both Python and Stata. In practice, we found that relying on a single programming environment can miss some correct matches for reasons tied to the underlying package behavior. Running both therefore helps us avoid these package-specific limitations and recover matches that the other method sometimes overlooks. In Python, we use a two-step process with token-set similarity to select candidates, followed by a stricter verifier, using the package rapidfuzz. We clean names by lowercasing, removing extra spaces and punctuation, dropping common suffixes, normalizing common ab- breviations, and excluding generic stopwords such as “hospital,” “medical,” and “health care.” We also incorporate location information. In Stata, we use reclink2 with cleaned names. Stata trimming is less granular, which yields a broader candidate set but reliably captures correct matches that Python can occasionally miss.

**Verification using location information** We merge Python and Stata candidates and apply location-based rules to confirm or reject links, then confirm exact or high-score matches with identical city/ZIP code/addresses and drop cross-state matches. This replicable rule set reduces case-by-case checks and minimizes false positives.

**Manual verification and matching** We manually check all verified fuzzy matches for con- sistency in names, locations, levels, and descriptions, and assigned a final verification flag. We then manually search unmatched entries or unverified candidates against AHA and CMS to recover additional links. A final team review produces source-specific linkage files for Levin, PitchBook, Preqin, Capital IQ, FactSet, and SDC.

All manual verification (at this and all other steps in the process) is done manually, not by AI or LLMs, and checked for consistency and accuracy by multiple individuals.

Table [A1](#_bookmark26) outlines the process to this point, which results in a separate, final list of deals in each source. The resulting AHA-linked files include three types of linkages: single-hospital deals, which map to one AHA hospital-ID; multi-hospital deals (collapsed before), which map to multiple hospital-IDs; and system-level deals, which map to a system-ID.

## Reconciliation Across Sources

To construct a unified PE–AHA dataset, we cannot simply append the AHA-linked files from each source, as many deals appear in multiple PE datasets. We therefore identify and reconcile

**Table A1:** Construction of a Deal List from a Single Source

**Step Purpose Key actions Deliverable**

1. Define universe Filter deals dataset to hospital targets (or start

from a broader set and apply keyword filtering)

1. Flag PE deals Use acquirer descriptions and PE tags to

identify PE involvement

1. Name expansion Recover explicit facility or system names for

aggregated records

1. Build match keys Create AHA and deal dataset name keys;

include level flag and locations

Hospital-target list

PE deal subset

Expanded target names Harmonized match keys

1. Python fuzzy match

Two-step string matching on cleaned names using rapidfuzz; incorporate locations

Candidate matches (Python)

1. Stata fuzzy match reclink2 on cleaned names to broaden

candidate set

1. Rule-based checks Confirm with address, ZIP or city; drop

cross-state matches

1. Manual audit Human review and supplemental search using AHA and CMS

Candidate matches (Stata)

Verified candidate links

Final Deal-AHA linkage

overlapping entries to ensure that each underlying transaction is represented only once in the final deal list. To construct a unified PE–AHA deal list, we combine information from all PE data sources through a multi-step consolidation procedure. First, we assemble deal-level metadata from each source (acquirer identities, investor groups, deal dates, deal types, descriptive fields, etc.) and harmonize their formats. Second, we stack all AHA-linked deal entries rather than merging them on facility identifiers and dates.[^3^](#_bookmark27) We then sort the stacked entries by AHA ID and by single-source deal-date information to create a chronological order that is conducive to manual review. Third, for each hospital or system, we manually assign an “order” number to entries that represent the same underlying transaction based on metadata similarity. Finally, these order numbers allow the dataset to be easily collapsed into a version where cross-source overlaps are identified and each transaction is represented once.

## Applying System-Level Deals to Hospitals

To obtain a hospital-level deal universe, we expand system-level transactions into their con- stituent hospitals. Automatic expansion is performed using the AHA hospital–system link in the year prior to the transaction: all hospitals listed under the matched system in that AHA

^3^A direct merge on hospital/system IDs and deal dates is overly rigid because sources frequently report different timing conventions (e.g., announcement vs. closing dates) or contain internal discrepancies; strict date matching can therefore easily treat two reports of the same transaction as separate deals.

vintage are included. Manual expansion is triggered in two situations: (i) when the automatic expansion yields *N* = 0 hospitals for the system, or (ii) when the number of affiliated hospitals implied by AHA is substantially different from the hospital counts reported in external deal sources. In these cases, we verify and recover the constituent hospitals using earlier AHA files, 10-K/8-K SEC filings, press releases, and local news reports.

## Manual Verification of Hospital Identifiers

We first link PE deal records to hospitals observed in the AHA. Most hospital characteristics and outcomes used in the literature are drawn from CMS-based datasets, such as HCRIS and HCAHPS, where facilities are identified by Medicare provider numbers. To bridge these sources, we mainly rely on AHA’s built-in AHA-CMS crosswalk, which maps AHA facility identifiers to CMS provider numbers (*mcrnum*). Because provider numbers are not strictly time-invariant, reflecting mergers, restructurings, rebranding, or closures, we construct the crosswalk at the hospital deal-year level, assigning each hospital the provider number that best corresponds to its operational identity in the acquisition year.

The AHA-CMS crosswalk is imperfect and has several recurring issues, including missing entries, differences in facility coverage across AHA and CMS, and inconsistencies in how facilities are defined across sources. To address these issues, we supplement the crosswalk with additional verification and manual reconciliation. For hospitals with partially missing provider numbers, we impute values from adjacent years when the same provider number consistently tracks the same hospital over time and verify these assignments using CMS Provider of Services files (POS). For hospitals lacking a valid crosswalk in all years, we manually recover provider numbers using hospital names and addresses and confirm matches against POS files.

A further challenge arises from timing mismatches across AHA, CMS, and deal-level sources, where ownership changes may be reflected one year earlier or later depending on reporting conventions. We address these timing issues by aligning identifier changes to the underlying transaction timing rather than relying mechanically on reported year-to-year changes. This approach avoids spurious hospital entry or exit and ensures a consistent classification of treated hospitals.

## Validation

Finally, we implement several steps to standardize variables across sources, reconcile inconsis- tencies, and create a research-ready deal- and hospital-level file. These improvements address discrepancies in PE classification, deal timing, investor reporting, and cross-dataset coverage.

### Harmonized Deal Classification

Because the six PE datasets use different conventions to flag PE involvement and occasionally disagree, we adopt a unified identification rule. A transaction is initially treated as a potential PE deal if any source classifies it as PE. We then verify acquirer status (PE-backed or not) using PitchBook investor data, company filings, and public information at the time of the transaction.

During the process of reviewing all PE-related transactions, we identified several deal types in which private equity participates. Subtype classification is anchored in PitchBook’s taxonomy of LBO/buyout, growth equity, and PIPE, which provides the most consistent labeling across sources. For deals that remain uncertain after applying these source-specific labels, we review the deal descriptions, investor information, and public disclosures to manually assign a deal type. This harmonized classification allows us to compare PE involvement across key attributes of ownership, control, and financing. Further details are provided in [Appendix C](#_bookmark33).

### Verified Deal Timing

Reported deal dates differ across sources (e.g., announcement vs. closing dates) and may conflict with information disclosed in filings or press releases. We therefore manually verify deal dates using SEC 10-K/8-K filings, local and national news reports, and press releases. None of the sources we used are created by AI or LLMs. We include links to all sources in our published dataset. We assign a final deal date using the following hierarchy:

- - - 1. Verified closing date (preferred),
      2. Verified announcement date,
      3. Maximum reported closing date across PE sources, and
      4. Maximum reported announcement date, if all else is missing This yields a consistent “treatment date” for empirical analysis.

We follow a similar process to identify “exit” dates (i.e., the date that the PE investment ends). We track exits only for LBO deals. We use the same sources to verify exit dates and include these dates in our published dataset to facilitate empirical analysis.

## Final Constructed Dataset Structure

Following the harmonization and linkage procedures described above, we produce a primary analysis-ready dataset. Each row is a hospital unit corresponding to a unique deal after recon- ciling overlaps across sources and consolidating system and hospital targets. This file contains

hospital identifiers, names and locations, deal level, transaction type, harmonized deal dates, and source links documenting the deal timing. Table [A2](#_bookmark28) details the key variables included in the dataset.

**Table A2:** Hospital-level master: core variables

**Variable Definition / Notes**

hospital id Unique and stable hospital identifier

hospital cms id CMS hospital identifier in the year of transaction

hospital name Hospital name

hospital city, hospital state,

hospital address, hospital zip

Hospital location

deal id Unique transaction identifier that is constant across hospitals acquired as part of the same deal

deal year Year the deal took place

deal system level Indicator for whether the deal was a system-level rather than

hospital-level deal

system name Name of the hospital system associated with the system-level deal

deal type Categories: LBO, growth equity, and PIPE

deal announcement date Date the transaction was publicly announced, when available.

deal closing date Transaction closing date, when available.

deal exit date Date of PE exit, when available (LBO only)

deal announcement link, deal closing link

Source URLs documenting the announcement and closing of the transaction, when available

# Appendix B Supplementary Tables and Figures

**Table B1:** Unique Hospital Counts by Deal Type

**An Observation is a Unique Hospital**

*Deal Type*

| LBO | 391 | 70.4% |
| --- | --- | --- |
| PIPE | 54 | 9.7% |
| Growth Equity | 140 | 25.2% |

*Note:* This table reports the counts of unique hospitals that had each type of deal. Note that each hospital may have multiple deal types, so these counts sum to greater than the total number of unique hospitals that had a PE acquisition (555). The final column reports the share of the 555 PE-acquired hospitals that had at least one of the relevant deal type.

**Table B2:** Comparison of PitchBook and Levin

PitchBook not Levin Levin not PitchBook

**Panel A: An Observation is a Hospital-Deal Pair**

| Hospital-Deal Count | 299 | 20 |
| --- | --- | --- |
| *Deal Level*  System Level | 93% | 15% |
| Hospital Level | 7% | 85% |
| *Deal Type*  LBO | 33% | 100% |
| PIPE | 49% | 0% |
| Growth Equity | 18% | 0% |
| **Panel B: An Observation is a Deal** | | |
| Deal Count | 42 | 19 |
| *Deal Level*  System Level | 48% | 11% |
| Hospital Level | 52% | 89% |
| *Deal Type*  LBO | 60% | 100% |
| PIPE | 12% | 0% |
| Growth Equity | 28% | 0% |

*Note:* Panel A reports characteristics of PE acquisitions of hospitals, where each observation represents a hospital-deal pair rather than a unique hospital. Panel B reports characteristics at the deal, rather than hospital, level. “System Level” deals include only deals involving the full hospital system. “Hospital Level” deals include both single-hospital and multi-hospital deals (e.g., five Miami HCA Hospitals).

**Figure B1:** Source Shares of Hospitals and Deals by Year


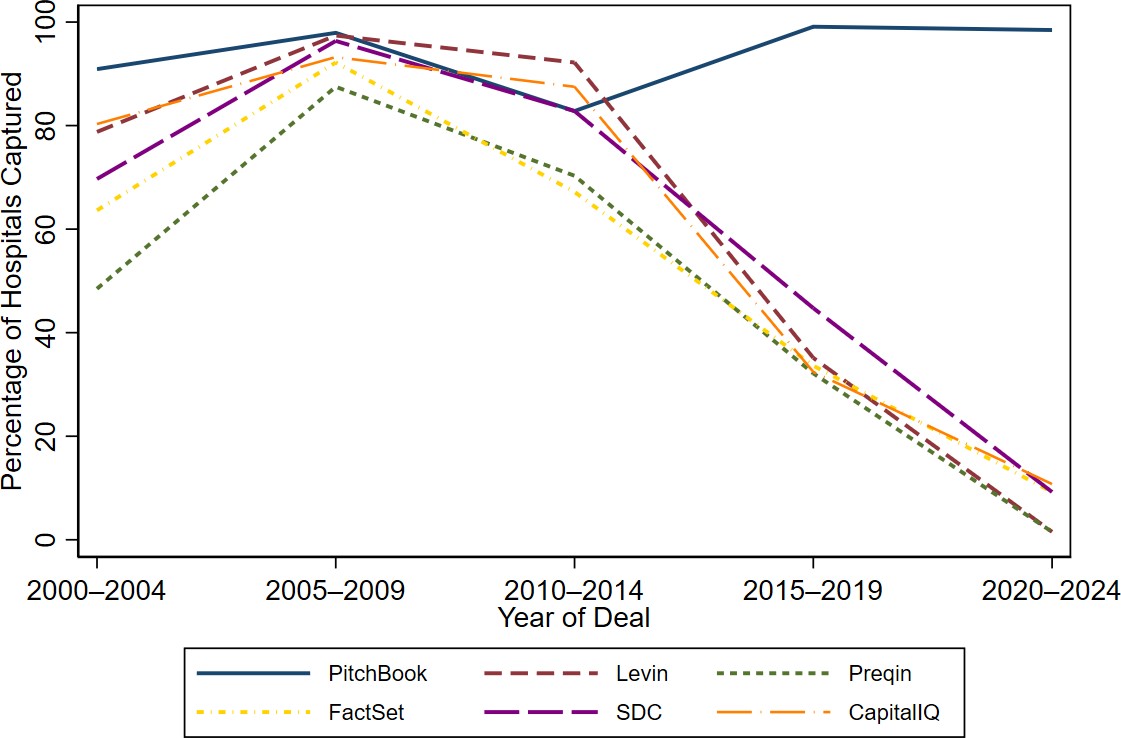


1. Hospitals


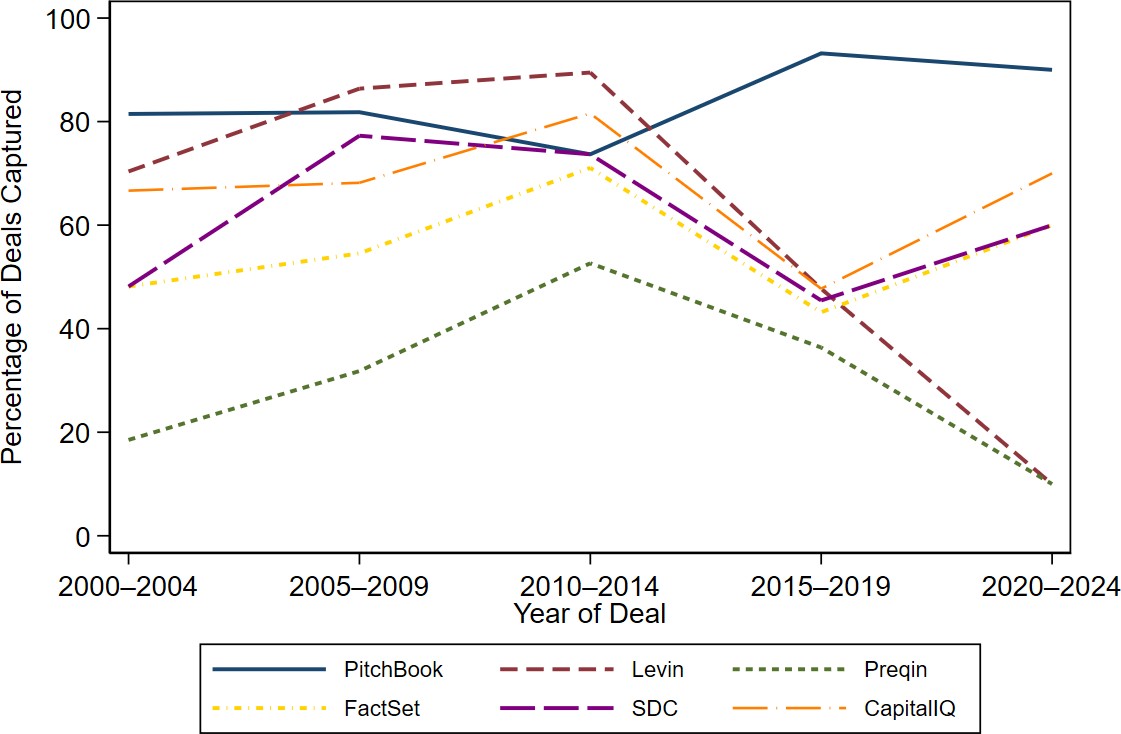


1. Deals

*Note:* These shares reflect the percentage of total hospital/deal entries in each year found in each source. They sum to greater than 100% as the same hospital/deal can be found in multiple sources.

# Appendix C PE Deal Classifications and Definitions

Private equity transactions in health care span several deal structures that differ in ownership stakes, operational control, financing mechanisms, and strategic intent. Although our primary classification relies on PitchBook, Preqin, SDC, FactSet, and Capital IQ, we unify definitions across datasets using standard private equity terminology and manual review. This appendix summarizes the main PE involvement types observed in our dataset and outlines their concep- tual differences. For the purposes of this article, we combine LBO, add-on, and other buyouts into one “LBO” category for our deal-type classification.

**Table C1:** Comparison of Private Equity Involvement Types Across Key Attributes

| **Deal Type** | **Ownership Acquired** | **Operational Control** | **Debt Financing** |
| --- | --- | --- | --- |
| LBO | 100% | Full control | Heavy leverage |
| PIPE | Partial/minority | Partial influence | Usually none |
| Growth Equity | Partial/minority | None | Sometimes |

## Definitions and Characteristics by Deal Type

**LBO (Leveraged Buyout)** An LBO occurs when a PE firm acquires controlling ownership, typically 100% of a hospital or system using substantial debt financing. The acquired entity’s cash flows are expected to service the debt, with the goal of operational restructuring and future value creation. Our LBO categorization includes add-on deals where an acquisition made by an existing PE-owned platform to expand the platform’s scale, service lines, or geographic footprint. It is also inclusive of management buyouts and secondary buyouts, which are LBOs of particular targets.

**PIPE (Private Investment in Public Equity)** In a PIPE deal, a PE firm purchases a minority equity stake in a publicly traded hospital company, gaining influence (but not control) through preferred shares, board rights, or strategic agreements.

**Growth Equity** Growth Equity is a minority or non-controlling investment used to fund development projects, service-line expansions, facility upgrades, or acquisitions by the hospital or system itself.

# Appendix D Identification and Data Construction in Prior Studies

This appendix documents how prior studies identify private equity (PE) acquisitions of hos- pitals, verify PE ownership and transaction closure, link ownership changes to hospital-level provider identifiers, and define analytic sample restrictions, including deal-type inclusion, hospital- type definitions, study periods, and panel requirements. Table [D1](#_bookmark35) summarizes these method- ological choices across the existing literature.

**Table D1:** How Studies Construct PE Deal Data

| **Paper** |  | **Identification of PE Deals** | **Verification** | **Sample Restrictions and Identifier Linkage** |
| --- | --- | --- | --- | --- |
| Bruch, Gondi, Song (2020)[^3^](#_bookmark6) | and | PE control defined as direct purchase, portfolio-company | SEC filings, press releases, hospital websites; PE status via | Non-PE to PE ^a^; ACH ^b^; 2005-2017; Linked to HCRIS |
|  |  | purchase, or majority | PitchBook; exposure = closure | and AHA |
|  |  | ownership of a health system |  |  |
| Bruch, Zeltzer, Song (2021)[^4^](#_bookmark7) | and | PE control defined as hospitals under private equity ownership | Not Specified | ACH; full-year HCRIS (2018) |
|  |  | in 2018 |  |  |
| Offodile et (2021)[^14^](#_bookmark16) | al. | LBOs of hospitals or health systems, inclusive of primary | Three-author independent verification; exclude incomplete | Non-PE to PE; ACH;  2003-2017; Linked to HCRIS |
|  |  | and add-on acquisitions | cases; exposure = ownership | and AHA |
|  |  |  | transfer; system deals |  |
|  |  |  | disaggregated via AHA *SYSID* |  |
|  |  |  | (year *t*) and validated using PE |  |
|  |  |  | firm and hospital 10-K filings. |  |
| Cerullo et al., Cerullo | | Primary and add-on LBOs | Cross-reference press, | Non-PE to PE; ACH; restricted |
| et al., Cerullo et al.  (2021, 2022, 2022)[^5^](#_bookmark8)^–^[^7^](#_bookmark10) | |  | newsletters, and trade media;  exclude ambiguous cases; Index year = transfer of control | time window; require complete  pre/post observation window; linked to CMS and AHA ^c^ |
| Liu (2022)[^13^](#_bookmark15) | | PE Buyouts | Cross-check across sources; | 2006-2019; Linked to CMS and |
|  | |  | infer exits via web/press when | AHA |
|  | |  | not reported |  |
| Kannan, Bruch, and | | First-time PE acquisition of a | Independently confirm | Non-PE to PE; ACH; |
| Song (2023)[^10^](#_bookmark13) | | previously non-PE hospital; | acquisitions via filings and press | 2010–2017; 1 pre- and 2 |
|  | | year assigned at closure; |  | post-acquisition years of |
|  | |  |  | Medicare claims data |
| Gao, Kim, and Sevilir | | Seed list = Cooper et al. | Manually verify *SYSID* changes | First event only (*>*5-year gap); |
| (2025)[^9^](#_bookmark12) | | (2019)[^19^](#_bookmark21) mergers (2001–2014); | using SDC, FactSet, Becker’s, | exclude *<*5-year repeats; |
|  | | extended to 2018 via AHA | news, and AHD; confirm PE | 2001–2018; general medical and |
|  | | *SYSID* flags; PE = firm or | status via Preqin, Capital IQ, | surgical hospitals; Linked to |
|  | | PE-owned roll-up | Becker’s | CMS and AHA |
| Diaz et al. (2025)[^8^](#_bookmark11) | | Non-PE hospital acquired by a | Independently confirmed via | Non-PE to PE; ACH and CAH; |
|  | | PE firm | public records | 2011–2020; 2 years Medicare |
|  | |  |  | claims pre/post acquisition |
| Bhatla et al. (2025)[^2^](#_bookmark5) | | Newly acquired by PE firms | Manually verified using official | Non-PE to PE; ACH; |
|  | |  | news releases, hospital websites, | 2010-2017; linked to HCAHPS |
|  | |  | and online searches | and AHA |
| Kannan et al. (2025)[^11^](#_bookmark14) | | Non–PE owned hospitals that underwent initial acquisition by | Not specified | Non-PE to PE; ACH; 1 pre- and 2 post-acquisition years of |
|  | | PE firms |  | Medicare claims data. |

*Note:* The table summarizes how each study constructed its PE deal data and applied analytic sample restric- tions, including deal-type inclusion (e.g., primary buyouts vs. all PE transactions), hospital-type definitions (e.g., short-term acute care), study periods, and pre/post panel requirements.

Abbreviations: AHA = American Hospital Association; HCRIS = Healthcare Provider Cost Reporting In- formation System; CMS = Centers for Medicare & Medicaid Services; AHD = American Hospital Directory; HCAHPS = Hospital Consumer Assessment of Healthcare Providers and Systems.

^a^ “Non-PE to PE” indicates whether treatment is defined as a transition from non–private equity ownership to private equity ownership. In most studies, this corresponds to a primary buyout or first-time acquisition of a previously non-PE-owned hospital, though terminology and operational definitions vary across papers.

^b^ ACH denotes short-term acute care hospitals as defined by CMS. Most studies restrict to this category. Some use AHA classifications instead, which may generate small differences in sample inclusion relative to CMS.

^c^ Study periods differ: 2006-–2015; 2005-–2014 (first acquisition retained if multiple); and 2001—2018 (*≥* 3 pre- and *≥*3 post-acquisition years required).

We report hospital counts in Table [2](#_bookmark3) to compare the coverage of our consolidated deal list to previous studies. This section of the appendix documents how these counts are calculated.

We classify each prior study according to six key data restrictions:

- - 1. Years of PE Deals Considered
    2. Type of PE Deals Considered
    3. Treatment of Multiple Deals Involving the Same Hospital
    4. Hospital Type
    5. Outcome Data
    6. Other Restrictions

Table [D2](#_bookmark36) summarizes these restrictions.

We apply these restrictions to our deal list to calculate the “Expanded” column in Table [2](#_bookmark3). While some studies place data availability restrictions on claims data, as shown in Table [D1](#_bookmark35), no restrictions are placed on other outcome data. Our calculations of these counts do not account for claims availability, as the use of claims is outside the scope of this article.

We apply only the year restrictions from these studies on our deal list to calculate the “Maximum” in Table [2](#_bookmark3). These counts are larger, as they include all deal types, allow for multiple deals involving the same hospital, and do not consider data availability.

Finally, we note that the hospital counts implied by our harmonized deal list are not directly comparable to those reported in Gao, Kim, and Sevilir (2025)[^9^](#_bookmark12) and Liu (2022)[^13^](#_bookmark15), as they include all hospital types, whereas we restrict our sample to CMS-designated short-term acute care hospitals. When a study uses the AHA definition of short-term acute care hospitals, we apply the AHA short-term acute care restriction within our CMS-designated short-term acute care sample, as our deal list does not include hospitals outside the CMS short-term acute care universe.

Please note that we are not claiming that the data from previous studies are faulty. Rather, we seek to highlight that differences in deal coverage among commercial databases result in different determinations of PE exposure. We hope that our use of six commercial deal databases and transparent data construction will help others overcome the major challenges in cleaning data and determining PE exposure.

**Table D2:** Data Restrictions Across the Literature

| **Study** | **Years** | **Deal Type** | **Multiple Deals** | **Hospital Type** | **Outcome Data** | **Other** |
| --- | --- | --- | --- | --- | --- | --- |
| Bhatla et al. (2025)[^2^](#_bookmark5) | 2010–2017 | Acquisitions*^a^* | First-Time | AHA | HCAHPS |  |
|  |  |  |  | Short-Term |  |  |
|  |  |  |  | Acute Care |  |  |
| Bruch, Gondi, and Song (2020)[^3^](#_bookmark6) | 2005-2017 | Purchases*^a^* | Not Explicit*^b^* | CMS  Short-Term | HCRIS,  Medicare |  |
|  |  |  |  | Acute Care | Claims |  |
| Bruch, Zeltzer, and Song (2021)[^4^](#_bookmark7) | 2018 | Under PE Control*^c^* |  | CMS  Short-Term |  |  |
|  |  |  |  | Acute Care |  |  |
| Cerullo et al. (2021)[^5^](#_bookmark8) | 2006–2015 | LBO, Add-On | Not Explicit*^b^* | CMS | HCRIS, AHA |  |
|  |  |  |  | Short-Term |  |  |
|  |  |  |  | Acute Care |  |  |
| Cerullo et al. (2022)[^6^](#_bookmark9) | 2005–2014 | Ownership Transfer*^a^* | First-Time | CMS  Short-Term | HCRIS |  |
|  |  |  |  | Acute Care |  |  |
| Cerullo et al. (2022)[^7^](#_bookmark10) | 2003–2015 | LBO, Add-On | First-Time | CMS | HCIRS, |  |
|  |  |  |  | Short-Term | Medicare |  |
|  |  |  |  | Acute Care | Claims |  |
| Diaz et al. (2025)[^8^](#_bookmark11) | 2013–2018*^d^* | Acquisitions*^a^* | First-Time | CMS | AHA, |  |
|  |  |  |  | Short-Term | Medicare |  |
|  |  |  |  | Acute Care, | Claims |  |
|  |  |  |  | CAH |  |  |
| Gao, Kim, and Sevilir (2025)[^9^](#_bookmark12) | 2001–2018 | Acquisitions*^a^* | Unique Hospitals | All | HCRIS,  Private |  |
|  |  |  |  |  | Claims |  |
| Kannan, Bruch, and Song (2023)[^10^](#_bookmark13) | 2010–2017 | Transfers*^a^* | Not Explicit*^b^* | CMS  Short-Term | Medicare Claims |  |
|  |  |  |  | Acute Care |  |  |
| Kannan et al. (2025)[^11^](#_bookmark14) | 2010–2017 | Acquisitions*^a^* | First-Time | CMS  Short-Term | HCRIS,  Medicare | ED or ICU |
|  |  |  |  | Acute Care | Claims |  |
| Liu (2022)[^13^](#_bookmark15) | 2006–2019 | Buyout, | Not Explicit*^b^* | All | None*^e^* |  |
|  |  | Growth |  |  |  |  |
|  |  | Equity |  |  |  |  |
| Offodile et al. (2021)[^14^](#_bookmark16) | 2003–2017 | LBO, Add-On | Not Explicit*^b^* | AHA  Short-Term | AHA, HCRIS |  |
|  |  |  |  | Acute Care |  |  |

*Note:* The table summarizes data restrictions used by each study. Our interpretation of these restrictions informs our calculations of the counts in Table [2](#_bookmark3)

Abbreviations: AHA = American Hospital Association; HCRIS = Healthcare Provider Cost Reporting Information System; CMS = Centers for Medicare & Medicaid Services; HCAHPS = Hospital Consumer Assessment of Healthcare Providers and Systems; ED = Emergency Department; ICU = Intensive Care Unit; LBO = Leveraged Buyout.

Our “buyout” deal type is inclusive of both LBO and add-on. “First-time” denotes that only initial PE investments are considered, meaning only the first deal is included for hospitals that multiple deals during the time period.

^a^ The terms “acquisitions”, “purchases”, “transfers”, and “ownership transfers” were all interpreted to refer to our “buyout” deal type.

^b^ Many studies do not explicitly state how multiple acquisitions are handled. Our interpretation of all such cases led us to consider only “first-time” acquisitions.

^c^ “Under PE control” is interpreted to include “buyout” deals where the PE investor has yet to exit the deal.

^d^ The time window 2013–2018 is not explicitly stated, but is inferred from the stated years of Medicare claims

and data restrictions on Medicare claims. 28

^e^ The counts referenced in Liu (2022)[^13^](#_bookmark15) come from analysis of exposure alone and do not rely on any outcome data.
